# 2,2′-Bithio­phene-3,3′-dicarbonitrile

**DOI:** 10.1107/S1600536812032503

**Published:** 2012-07-25

**Authors:** J. Josephine Novina, G. Vasuki, Durai Karthik, K. R. Justin Thomas

**Affiliations:** a Department of Physics, Idhaya College for Women, Kumbakonam-1, India; b Department of Physics, Kunthavai Naachiar Government Arts College (W) (Autonomous), Thanjavur-7, India; c Organic Materials Lab, Department of Chemistry, Indian Institute of Technology Roorkee, Roorkee 247 667, India

## Abstract

The complete mol­ecule of the title compound, C_10_H_4_N_2_S_2_, is generated by an inversion center situated at the mid-point of the bridging C—C bond. The bithio­phene ring system is planar [maximum deviation = 0.003 (2) Å] and the central C—C bond length is 1.450 (2) Å. There are no significant inter­molecular inter­actions in the crystal structure, which is stabilized by van der Waals inter­actions.

## Related literature

For the importance of bithio­phene derivatives, see: Katz *et al.* (1995 ▶). For their applications, see: Deng *et al.* (2011 ▶); Thomas *et al.* (2008 ▶). For background to the title compound, see: Demanze *et al.* (1996 ▶); Pletnev *et al.* (2002 ▶); For related structures, see: Benedict *et al.* (2004 ▶); Huang & Li (2011 ▶); Pelletier *et al.* (1995 ▶); Li & Li (2009 ▶); Teh *et al.* (2012 ▶). For thio­phene C—S bond lengths, see: Howie & Wardell (2006 ▶). For the normal bonding picture for bithio­phene, see: Khan *et al.* (2004 ▶).
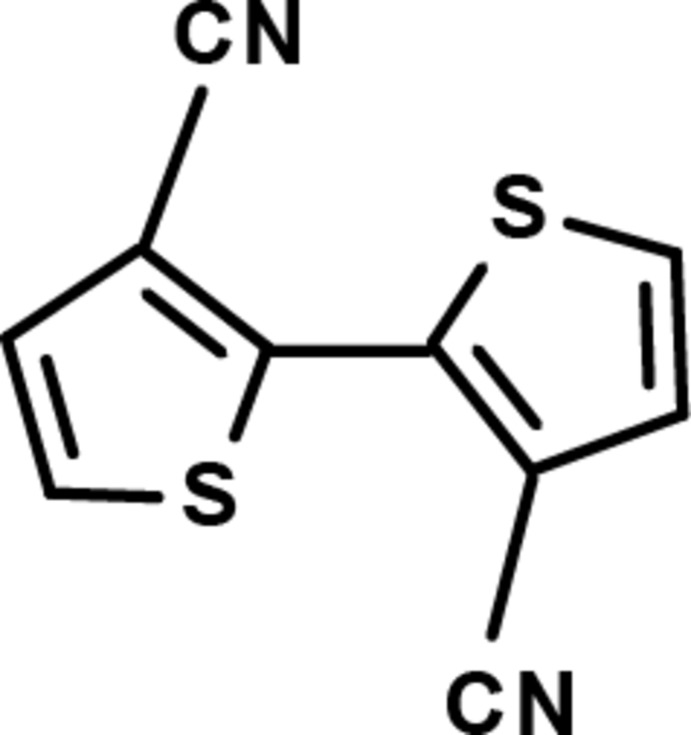


## Experimental

### 

#### Crystal data

C_10_H_4_N_2_S_2_
*M* *_r_* = 216.27
Monoclinic, 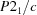

*a* = 3.9084 (1) Å
*b* = 9.8832 (4) Å
*c* = 12.0091 (5) Å
β = 93.900 (2)°
*V* = 462.81 (3) Å^3^
*Z* = 2
Mo *K*α radiation
μ = 0.53 mm^−1^
*T* = 293 K
0.30 × 0.20 × 0.20 mm

#### Data collection

Bruker Kappa APEXII CCD diffractometer
Absorption correction: multi-scan (*SADABS*; Bruker, 2004 ▶) *T*_min_ = 0.881, *T*_max_ = 0.900
6689 measured reflections
1802 independent reflections
1409 reflections with *I* > 2σ(*I*)
*R*_int_ = 0.019

#### Refinement

*R*[*F* ^2^ > 2σ(*F* ^2^)] = 0.034
*wR*(*F* ^2^) = 0.105
*S* = 1.05
1802 reflections
64 parameters
H-atom parameters constrained
Δρ_max_ = 0.36 e Å^−3^
Δρ_min_ = −0.20 e Å^−3^

### 

Data collection: *APEX2* (Bruker, 2004 ▶); cell refinement: *APEX2* and *SAINT* (Bruker, 2004 ▶); data reduction: *SAINT* and *XPREP* (Bruker, 2004 ▶); program(s) used to solve structure: *SHELXS97* (Sheldrick, 2008 ▶); program(s) used to refine structure: *SHELXL97* (Sheldrick, 2008 ▶); molecular graphics: *ORTEP-3* (Farrugia, 1997 ▶); software used to prepare material for publication: *PLATON* (Spek, 2009 ▶).

## Supplementary Material

Crystal structure: contains datablock(s) I, global. DOI: 10.1107/S1600536812032503/su2475sup1.cif

Structure factors: contains datablock(s) I. DOI: 10.1107/S1600536812032503/su2475Isup2.hkl

Supplementary material file. DOI: 10.1107/S1600536812032503/su2475Isup3.cml

Additional supplementary materials:  crystallographic information; 3D view; checkCIF report

## Figures and Tables

**Fig. 1.**
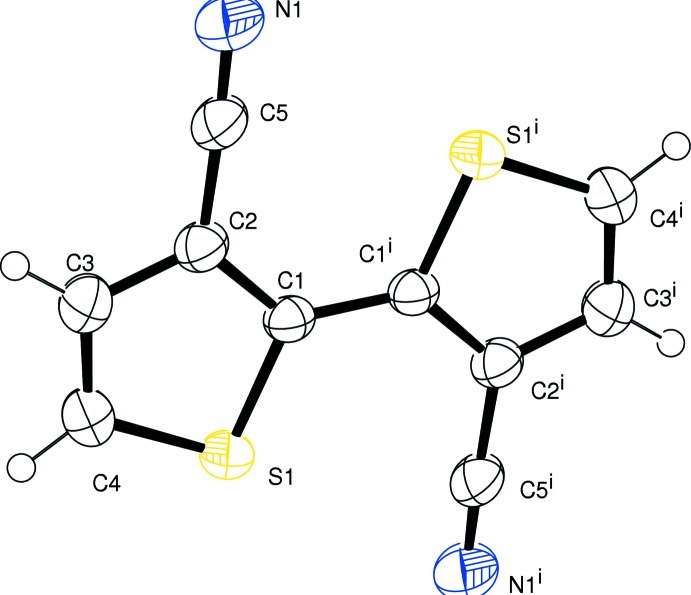
The molecular structure of the title molecule showing the atom numbering. the displacement ellipsoids are drawn at the 50% probability level.

## supplementary crystallographic information

### Comment

Bithiophene derivatives are important compounds in the synthesis of oligothiophenes and polythiophenes which have attracted attention as materials showing interesting characteristics as conducting, nonlinear optical (NLO), and liquid crystalline materials (Katz *et al.*, 1995). Oligothiophenes and their derivatives are useful precursors for the construction of organic materials suitable for application in electronic devices (Deng *et al.*, 2011; Thomas *et al.*, 2008) and the presence of an electron-withdrawing cyano group may offer a route to tune the electronic properties of the resulting materials. Our interest in these derivatives has led us to prepare the title compound which is known in the literature (Pletnev *et al.*, 2002; Demanze *et al.*, 1996) but was obtained as a side product during the attempted synthesis of other derivatives. We herein report on the direct synthesis and the crystal structure of the title compound.

The asymmetric unit of the title compound, comprises half a molecule with the full molecule generated by a crystallographic centre of inversion (Fig. 1). The bithiophene unit is planar to within 0.003 (2) Å. Within the bithiophene unit, the C1—C2 and C3—C4 bond-lengths [1.3838 (16) and 1.3487 (19) Å, respectively] are significantly shorter than bond C2—C3, 1.4173 (19) Å. This is consistent with the normal bonding picture for bithiophene (Khan *et al.*, 2004).

One feature of the molecule is the difference between the S1—C1 and S1—C4 bond lengths [1.724 (1) and 1.700 (1) Å, respectively]. Howie and Wardell (2006) have noted a similar disparity in the S—C bond lengths. This generally agrees with those values found for related structures, such as 2,2'-[2,5-Bis(hexyloxy)-1,4-phenylene]-dithiophene (Teh *et al.*, 2012) and 3,3',5,5'-Tetrabromo-2,2'-bithiophene (Li & Li, 2009).

The carbonitrile chain is almost linear, with the N1-C5-C2 bond angle being 177.43 (15)°. The geometric parameters are comparable with those observed in the related structures 3,3'-Bis(octyloxy)-2,2'-bithiophene at 195 K (Pelletier *et al.*, 1995), 2,2'-(3,3'-Dihexyl-2,2'-bithiophene-5,5'-diyl) bis(4,4,5,5-tetramethyl-1,3,2-dioxa-borolane) [Huang & Li, 2011] and 3,3'-Didecyl-5,5,-bis(4-phenylquinolin-2-yl)-2,2'-bithienyl (Benedict *et al.*, 2004).

There are no significant hydrogen-bonding interactions in the crystal structure, which is stabilized by van der Waals interactions.

### Experimental

Copper(I) cyanide (5.17 g, 57.72 mmol) was added to a solution of 3,3'-dibromo-2, 2'-bithiophene (6.23 g, 19.24 mmol) in 50 ml of DMF. This mixture was heated at 423 K for 32 h under nitrogen atmosphere. After cooling to room temperature, 50 ml of aqueous ammonia solution was added and allowed to stir for 4 h at room temperature. It was extracted with ethyl acetate and the combined organic layer washed with 3× 100 ml of water and dried over anhydrous sodium sulfate. On vacuum evaporation it produced a crude solid which was purified by column chromatography on silica gel using 4:1 mixture of hexanes and ethylacetate as eluant, to give a pale yellow solid; Yield 1.66 g (40%). Yellow block-like crystals were grown from an ethylacetate/hexane (1:4) mixture (M.p. 477 K). Spectroscopic data for the title compound are given in the archived CIF.

### Refinement

All the H atoms were positioned geometrically and refined using a riding model: C—H = 0.93 Å with *U*_iso_(H) = 1.2*U*_eq_(C).

### Crystal data

**Table:**

C_10_H_4_N_2_S_2_	*Z* = 2
*M**_r_* = 216.27	*F*(000) = 220
Monoclinic, *P*2_1_/*c*	*D*_x_ = 1.552 Mg m^−^^3^
Hall symbol: -P 2ybc	Mo *K*α radiation, λ = 0.71073 Å
*a* = 3.9084 (1) Å	θ = 2.7–33.5°
*b* = 9.8832 (4) Å	µ = 0.53 mm^−^^1^
*c* = 12.0091 (5) Å	*T* = 293 K
β = 93.900 (2)°	Block, yellow
*V* = 462.81 (3) Å^3^	0.30 × 0.20 × 0.20 mm

### Data collection

**Table:**

Bruker Kappa APEXII CCD diffractometer	1802 independent reflections
Radiation source: fine-focus sealed tube	1409 reflections with *I* > 2σ(*I*)
Graphite monochromator	*R*_int_ = 0.019
ω and φ scan	θ_max_ = 33.5°, θ_min_ = 2.7°
Absorption correction: multi-scan (*SADABS*; Bruker, 2004)	*h* = −5→5
*T*_min_ = 0.881, *T*_max_ = 0.900	*k* = −15→14
6689 measured reflections	*l* = −18→18

### Refinement

**Table:**

Refinement on *F*^2^	Primary atom site location: structure-invariant direct methods
Least-squares matrix: full	Secondary atom site location: difference Fourier map
*R*[*F*^2^ > 2σ(*F*^2^)] = 0.034	Hydrogen site location: inferred from neighbouring sites
*wR*(*F*^2^) = 0.105	H-atom parameters constrained
*S* = 1.05	*w* = 1/[σ^2^(*F*_o_^2^) + (0.0536*P*)^2^ + 0.0819*P*] where *P* = (*F*_o_^2^ + 2*F*_c_^2^)/3
1802 reflections	(Δ/σ)_max_ = 0.001
64 parameters	Δρ_max_ = 0.36 e Å^−^^3^
0 restraints	Δρ_min_ = −0.20 e Å^−^^3^

### Special details

**Table:**

Experimental. Spectroscopic data for the title compund:IR (KBr, cm^-1^) 2221.0 (ν C≡N); ^1^H NMR (CDCl3, 500.13 MHz) δ 7.37 (d, J = 5.36 Hz, 2H),7.53 (d, J = 5.36 Hz, 2H); ^13^C NMR (CDCl3, 125.75 MHz); δ 110.0, 114.5, 128.3, 130.4, 141.0 p.p.m.
Geometry. All e.s.d.'s (except the e.s.d. in the dihedral angle between two l.s. planes) are estimated using the full covariance matrix. The cell e.s.d.'s are taken into account individually in the estimation of e.s.d.'s in distances, angles and torsion angles; correlations between e.s.d.'s in cell parameters are only used when they are defined by crystal symmetry. An approximate (isotropic) treatment of cell e.s.d.'s is used for estimating e.s.d.'s involving l.s. planes.
Refinement. Refinement of *F*^2^ against ALL reflections. The weighted *R*-factor *wR* and goodness of fit *S* are based on *F*^2^, conventional *R*-factors *R* are based on *F*, with *F* set to zero for negative *F*^2^. The threshold expression of *F*^2^ > σ(*F*^2^) is used only for calculating *R*-factors(gt) *etc*. and is not relevant to the choice of reflections for refinement. *R*-factors based on *F*^2^ are statistically about twice as large as those based on *F*, and *R*- factors based on ALL data will be even larger.

### Fractional atomic coordinates and isotropic or equivalent isotropic displacement parameters (Å^2^)

**Table:**

	*x*	*y*	*z*	*U*_iso_*/*U*_eq_	
S1	0.28852 (8)	0.68019 (3)	0.41382 (2)	0.04106 (12)	
C1	0.4855 (3)	0.57190 (12)	0.51077 (9)	0.0320 (2)	
C2	0.6002 (3)	0.64524 (13)	0.60413 (9)	0.0367 (2)	
C5	0.7804 (4)	0.59005 (15)	0.70133 (10)	0.0439 (3)	
C4	0.3580 (4)	0.81738 (13)	0.49772 (12)	0.0475 (3)	
H4	0.2877	0.9046	0.4781	0.057*	
C3	0.5265 (4)	0.78545 (14)	0.59594 (11)	0.0458 (3)	
H3	0.5872	0.8480	0.6517	0.055*	
N1	0.9252 (4)	0.55103 (16)	0.77997 (11)	0.0630 (4)	

### Atomic displacement parameters (Å^2^)

**Table:**

	*U*^11^	*U*^22^	*U*^33^	*U*^12^	*U*^13^	*U*^23^
S1	0.0472 (2)	0.03822 (18)	0.03623 (18)	0.00130 (11)	−0.00859 (12)	0.00367 (11)
C1	0.0314 (4)	0.0354 (5)	0.0287 (5)	−0.0021 (4)	−0.0009 (3)	0.0025 (4)
C2	0.0394 (6)	0.0387 (6)	0.0313 (5)	−0.0030 (5)	−0.0020 (4)	−0.0006 (4)
C5	0.0518 (7)	0.0431 (6)	0.0354 (6)	−0.0063 (5)	−0.0073 (5)	−0.0029 (5)
C4	0.0567 (8)	0.0339 (6)	0.0507 (7)	0.0022 (5)	−0.0053 (6)	0.0005 (5)
C3	0.0554 (8)	0.0380 (6)	0.0429 (7)	−0.0015 (5)	−0.0044 (5)	−0.0048 (5)
N1	0.0802 (10)	0.0599 (8)	0.0454 (6)	−0.0049 (7)	−0.0218 (6)	0.0021 (6)

### Geometric parameters (Å, º)

**Table:**

S1—C4	1.7000 (14)	C2—C5	1.4298 (16)
S1—C1	1.7240 (11)	C5—N1	1.1351 (17)
C1—C2	1.3838 (16)	C4—C3	1.3487 (19)
C1—C1^i^	1.450 (2)	C4—H4	0.9300
C2—C3	1.4173 (19)	C3—H3	0.9300
			
C4—S1—C1	92.80 (6)	N1—C5—C2	177.43 (15)
C2—C1—C1^i^	129.27 (13)	C3—C4—S1	112.37 (10)
C2—C1—S1	109.09 (9)	C3—C4—H4	123.8
C1^i^—C1—S1	121.63 (11)	S1—C4—H4	123.8
C1—C2—C3	113.76 (11)	C4—C3—C2	111.98 (12)
C1—C2—C5	125.17 (12)	C4—C3—H3	124.0
C3—C2—C5	121.07 (11)	C2—C3—H3	124.0

Symmetry code: (i) −*x*+1, −*y*+1, −*z*+1.
